# Knee Osteoarthritis: A Review of Pathogenesis and State-Of-The-Art Non-Operative Therapeutic Considerations

**DOI:** 10.3390/genes11080854

**Published:** 2020-07-26

**Authors:** Dragan Primorac, Vilim Molnar, Eduard Rod, Željko Jeleč, Fabijan Čukelj, Vid Matišić, Trpimir Vrdoljak, Damir Hudetz, Hana Hajsok, Igor Borić

**Affiliations:** 1 St. Catherine Specialty Hospital, 49210 Zabok/10000 Zagreb, Croatia; vilim.molnar@svkatarina.hr (V.M.); eduard.rod@svkatarina.hr (E.R.); zeljko.jelec@svkatarina.hr (Ž.J.); fabijan.cukelj@svkatarina.hr (F.Č.); vid.matisic@svkatarina.hr (V.M.); trpimir.vrdoljak@svkatarina.hr (T.V.); ortohud@gmail.com (D.H.); hana.hajsok@gmail.com (H.H.); igor.boric@svkatarina.hr (I.B.); 2 Eberly College of Science, The Pennsylvania State University, University Park, State College, PA 16802, USA; 3 The Henry C. Lee College of Criminal Justice and Forensic Sciences, University of New Haven, West Haven, CT 06516, USA; 4 Medical School, University of Split, 21000 Split, Croatia; 5 School of Medicine, Faculty of Dental Medicine and Health, University “Josip Juraj Strossmayer”, 31000 Osijek, Croatia; 6 School of Medicine, JJ Strossmayer University of Osijek, 31000 Osijek, Croatia; 7 Medical School, University of Rijeka, 51000 Rijeka, Croatia; 8 Medical School REGIOMED, 96 450 Coburg, Germany; 9 Medical School, University of Mostar, 88000 Mostar, Bosnia and Herzegovina; 10 Department of Nursing, University North, 48 000 Varaždin, Croatia; 11 Department of Orthopedics, Clinical Hospital “Sveti Duh”, 10000 Zagreb, Croatia; 12 Medical School, University of Zagreb, 10000 Zagreb, Croatia

**Keywords:** knee osteoarthritis, cytokines, epigenomics, platelet-rich plasma, mesenchymal stem cells, genetic therapy, phenotype

## Abstract

Being the most common musculoskeletal progressive condition, osteoarthritis is an interesting target for research. It is estimated that the prevalence of knee osteoarthritis (OA) among adults 60 years of age or older is approximately 10% in men and 13% in women, making knee OA one of the leading causes of disability in elderly population. Today, we know that osteoarthritis is not a disease characterized by loss of cartilage due to mechanical loading only, but a condition that affects all of the tissues in the joint, causing detectable changes in tissue architecture, its metabolism and function. All of these changes are mediated by a complex and not yet fully researched interplay of proinflammatory and anti-inflammatory cytokines, chemokines, growth factors and adipokines, all of which can be measured in the serum, synovium and histological samples, potentially serving as biomarkers of disease stage and progression. Another key aspect of disease progression is the epigenome that regulates all the genetic expression through DNA methylation, histone modifications, and mRNA interference. A lot of work has been put into developing non-surgical treatment options to slow down the natural course of osteoarthritis to postpone, or maybe even replace extensive surgeries such as total knee arthroplasty. At the moment, biological treatments such as platelet-rich plasma, bone marrow mesenchymal stem cells and autologous microfragmented adipose tissue containing stromal vascular fraction are ordinarily used. Furthermore, the latter two mentioned cell-based treatment options seem to be the only methods so far that increase the quality of cartilage in osteoarthritis patients. Yet, in the future, gene therapy could potentially become an option for orthopedic patients. In the following review, we summarized all of the latest and most important research in basic sciences, pathogenesis, and non-operative treatment.

## 1. Introduction

Osteoarthritis (OA) is the most common progressive musculoskeletal condition that can affect joints, but it mainly affects the hips and knees as predominant weight-bearing joints [1,2,3]. Knee osteoarthritis is characterized by structural modifications to primarily articular cartilage and the subchondral bone, but also Hoffa’s fat pad, synovia, ligaments and muscles, leading to the concept of observing OA as a whole joint disease [4,5,6,7].

Because of the higher prevalence of asymptomatic OA, it is approximated that 250 million people all over the world suffer from OA [8,9]. The prevalence of knee OA increased significantly over the last decades and continues to rise, partially because of the increasing prevalence of obesity and other risk factors, but also independently, of other causes [10]. It is estimated that the prevalence of knee OA among adults 60 years of age or older is approximately 10% in men and 13% in women [11]. According to GBD 2015 Disease and Injury Incidence and Prevalence Collaborators, approximately 85% of the burden of osteoarthritis worldwide is connected with knee OA [12]. This is also seen by a rise in prevalence of knee OA of 32.7% from 2005 to 2015, making OA one of the leading causes of global years lived with disability (YLD) [12]. OA causes an annual economic burden of at least USD 89.1 billion—which is between 1% and 2.5% of the gross domestic product in high-income countries, with knee and hip joint replacements as the majority of that cost [9,12]. Furthermore, after low-back pain, osteoarthritis is the second leading musculoskeletal disorder in Disability Adjusted Life Years (DALYs) calculation in the elderly population [13].

Age, previous knee injuries, but also obesity (increased body mass index (BMI)), joint malalignment and instability that result in increased mechanical stress are all strong risk factors for the development of knee OA [14,15,16]. Repetitive actions, such as often kneeling and heavy lifting, together with professional sports activities, such as long-distance running, football, handball and hockey are associated with a higher risk of developing OA, due to more frequent injuries, causing cartilage defects, meniscal and anterior cruciate ligament (ACL) tears [17,18,19,20].

Physical inactivity is also another important contributor to the increasing prevalence of OA, causing higher susceptibility to knee damage due to less stable and weaker joints [20]. However, the weakness of knee extensor muscles seems to be a weak risk factor, compared to previous knee injuries [8].

Men are less likely to develop OA than women, making sex one of the risk factors associated with OA development [8]. Narrower femurs, thinner patellae, greater angles of quadriceps and differences in the size of tibial condyles make women’s knee anatomy different from men’s, leading to different kinematics, which influences female sex to be more likely to develop OA, ultimately leading to a higher prevalence of OA in women [21,22].

Studies have shown that there is a connection between OA and a slightly increased risk of developing cardiovascular and atherosclerosis-related diseases [23,24,25]. In addition, people with lower-limb OA are more likely to develop depressive symptoms due to chronic pain, as the most frequent and the most severe consequence of OA [16]. As a leading cause of depressive episodes, chronic pain causes a vicious cycle in which pain limits physical activity and physical inactivity contributes to greater knee pain and weight gain [16,20]. Unquestionably, OA is affecting people’s mental health and impacting the odds of suicidal ideas as well, which makes OA not just an economic, but also a major social, burden [16,26]. As a chronic disease with pain as the dominant symptom, pain management and lifestyle changes are insufficient, and OA remains challenging to treat. Joint replacement surgery is the only option left in end-stage disease to increase the quality of life in cases where conventional symptomatic treatment did not provide satisfactory results, making knee arthroplasty in OA as a revolutionary operation and a defeat of orthopedics, medicine and science at the same time [8]. However, secondary prevention and recent therapeutic measures including intra-articular applications of corticosteroid injections, hyaluronic acid injections, platelet-rich plasma (PRP) or autologous micro-fragmented adipose tissue with stromal vascular fraction may slow down the existing condition.

Genetics plays an important role in the pathogenesis of OA, as observed in 40% to 80% of the hip or hand OA, but significantly less in knee OA [8]. To date, 90 genetic risk loci for the development of OA have been identified using genome-wide association studies (GWAS) but a majority of those have low effect sizes [27]. Studies have shown that apart from the genetic risk loci, epigenetic mechanisms have a substantial impact on OA pathogenesis and progression [27,28]. Moreover, there are geographical and ethnic differences in OA prevalence. African-Americans are more likely to develop symptomatic knee OA in comparison to other races, whereas hip OA prevalence is low in Asian and Oriental populations [5,8].

Knowing that OA is a progressive condition, it is of great importance to assess for early signs of OA. This can be done by screening patient-reported outcomes, such as pain, function and quality of life, clinical findings such as joint tenderness and crepitus, objective measures of physical activity, and various imaging modalities, such as magnetic resonance imaging, along with biochemical markers [29].

The purpose of this review is to highlight recent studies of OA pathophysiology, imaging and state-of-the-art treatment methods, including research on prevalence, potential risk factors and future joint regeneration strategies. In this critical insight, we will endeavor to answer the majority of asked questions and provide up-to-date information on the topics mentioned above.

## 2. Pathogenesis and Histology—Osteoarthritis as a Whole Joint Disease

The approach to OA changed a lot throughout history. At first, it was thought that OA is a disease of cartilage. Later, the perception was replaced by an idea that subchondral bone is also affected, but today it is known that all the tissues in or around the joint are influenced by the disease, leading to the concept of OA as a whole joint disease.

### 2.1. Articular Cartilage

**Figure 1:**
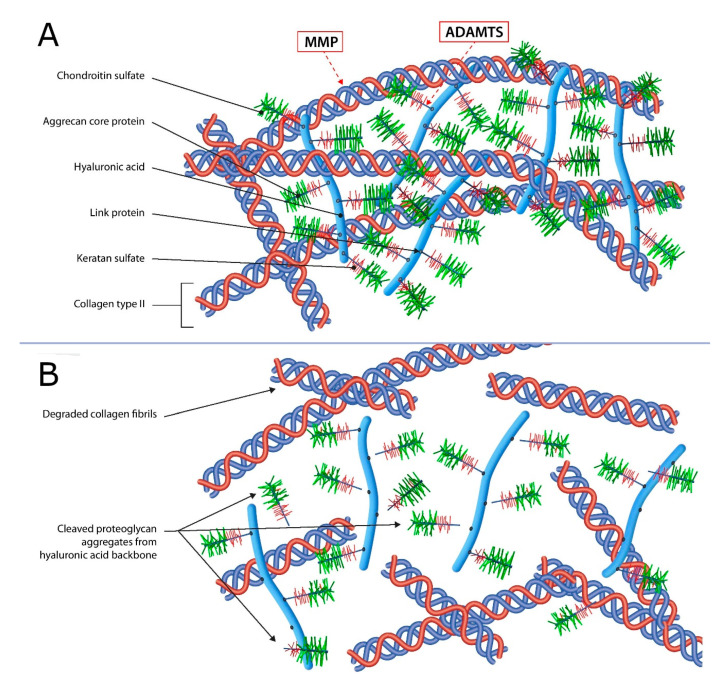
Schematic representation of cartilage extracellular matrix and its changes in osteoarthritis. (**A**) A healthy network of proteoglycan aggregates entangled with type II collagen fibres is seen. matrix metalloproteinases (MMPs) and a disintegrin and metalloproteinase with thrombospondin motifs (ADAMTS) cleavage sites are represented by red arrows; (**B**) Cartilage matrix changes in osteoarthritis defined by degradation of proteoglycans and cleavage of type II collagen fibres by ADAMTS and MMPs, respectively.

Articular cartilage (AC) is avascular, alymphatic, and aneural tissue with chondrocytes as the only cell type in the cartilage tissue [5,30,31]. Besides chondrocytes, AC is formed by the extracellular matrix (ECM), which is composed of water (more than 70%) and organic components such as type II collagen, aggrecan, other proteoglycans (decorin, biglycan, and fibromodulin), collagens (types III, VI, IX, XI, etc., collagens), glycosaminoglycans and glycoproteins [5,30,32]. Proteoglycan aggregates, built of negatively charged glycosaminoglycans (keratan sulfate and chondroitin sulfate) bound to the aggrecan core protein, that is linked with hyaluronic acid backbone, together with other matrix components, are entrapped in a network of cross-linked type II collagen fibrils, as portrayed in Figure 1 [31]. Premature termination codon on aggrecan mRNA affects cartilage development, while failure in aggrecan production may have a secondary effect on type II collagen, suggesting a possible extracellular matrix/type II collagen feedback regulation [33,34,35]. Although type II collagen and aggrecan are the most common proteins in the cartilage matrix, there is a distinct difference in the matrix structure around chondrocytes, where other proteins such as collagen VI, fibromodulin and matrilin 3 form the pericellular matrix [36]. All cartilage components are synthesized by chondrocytes, which play a key role in maintaining the cartilaginous environment by balancing the production of ECM components and its degrading enzymes, providing minimal and balanced turnover between anabolic and catabolic processes [37]. AC metabolism is stimulated by mechanical loading, detected by mechanoreceptors on the cell surface [38]. Through the process of mechanotransduction, mechanical signals modulate the biochemical activity of chondrocytes, inducing the biosynthesis of molecules to preserve the integrity of the tissue. Surface mechanoreceptors include mechanosensitive ion channels and integrins. Integrins are transmembrane proteins that activate internal cell signaling by binding chemical molecules, such as cytokines and growth factors [39]. The activation of these mechanoreceptors initiates intracellular signaling cascades, leading to the tissue remodeling process. Furthermore, biomechanical stimulus generated by dynamic compression during moderate exercise can reduce the synthesis of proteolytic enzymes, regulate the metabolic balance, and prevent the progression of cartilage damage [38]. The importance of proper mechanical loading is demonstrated by the fact that insufficient biomechanical stimuli, such as immobilization, can lead to reduced thickness (>10%) and softening of AC in the knee joint, in the absence of normal joint loading [40]. Conversely, excessive mechanical loading leads to a quantitative imbalance between anabolic and catabolic activity, resulting in the depletion of matrix components and, due to lack of AC regenerative capacity, leads to irreversible destruction, thus making it the most apparent triggering cause of OA [38].

**Figure 2:**
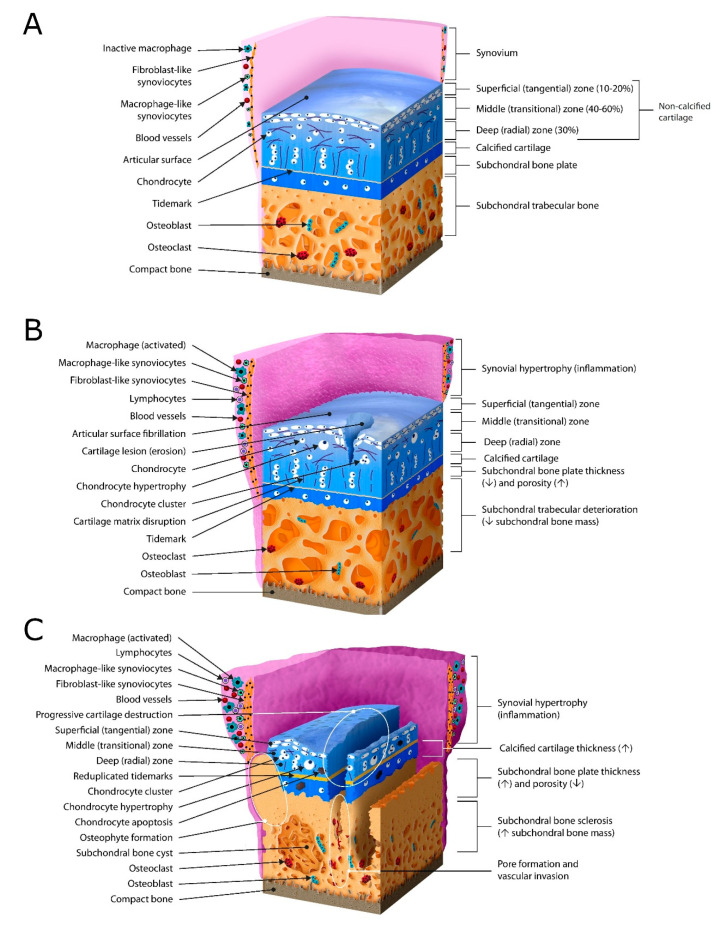
Microarchitectural and histologic changes of articular cartilage, subchondral bone and synovium in osteoarthritis. (**A**) Representation of normal joint structure and normal histologic state of the main tissue involved in osteoarthritis; (**B**) Early-stage osteoarthritis changes in articular cartilage, subchondral bone and synovium. Shallow cartilage erosions of the superficial tangential zone and middle transitional zone causing disruption of the extracellular matrix can be seen. Secondary chondrocyte hypertrophy and clustering is present as well. Changes to other joint tissues, mainly the reduction in subchondral bone mass, synovial thickening and inflammatory cells (lymphocytes) migration, together with changes in cellular activity and count, are depicted. (**C**) Late-stage osteoarthritis changes of involved tissues. Full-thickness cartilage erosions reaching the subchondral bone, chondrocyte apoptosis, subchondral bone sclerosis, osteophyte and subchondral cysts formation alongside vascular infiltration are shown. Further synovial thickening with immune cells infiltration and increased vascularization is also seen.

AC can be divided into four layers: superficial (tangential) zone, middle (transitional) zone, deep (radial) zone, and a highly mineralized zone of calcified cartilage. This calcified zone is separated from the unmineralized upper cartilage layers by a histologically defined zone called tidemark and divides the cartilage from the underlying subchondral bone [5,31,32]. The layers are characterized by chondrocyte shape and positioning, as well as collagen fibrils’ orientation [41,42,43,44,45]. In the superficial zone, chondrocytes are disk-shaped, and collagen fibrils are oriented horizontally. Collagen fibrils in the middle zone are diagonally directed and round-shaped chondrocytes are located randomly. The deep zone is defined by vertical columns of chondrocytes together with radially aligned collagen fibrils. In calcified cartilage, collagen fibrils are arranged perpendicular to the articular surface [46]. The population of chondrocytes is scarce in this zone. They are roundly shaped, without specific positioning [30]. A detailed representation can be seen in Figure 2.

Of the changes observed in OA, the cartilage matrix appears to be altered early and undergoes several pathological changes. As mechanoreceptors sense physical forces, chondrocytes answer with an adaptation of their metabolic activity [47]. One of the first changes, that results from excessive mechanical loading, is an increase of water content in the superficial zone of articular cartilage, with the loss of glycosaminoglycans and proteoglycan degradation. As OA advances, a process known as matrix swelling expands to the deep zone [48].

In early-stage OA, when macroscopic joint changes are not yet seen, the cartilage matrix goes through changes with the help of degrading enzymes. During this stage, aggrecanases of ADAMTS (a disintegrin and metalloproteinase with thrombospondin motifs), particularly ADAMTS-4 and ADAMTS-5, cleave the aggrecan core protein from the hyaluronic acid backbone seen in Figure 1 [49]. The cleavage site is located beneath the glycosaminoglycan (GAG)-rich part of the aggrecan, which leads to their detachment from the proteoglycan aggregate. Since GAG molecules are responsible for osmotic properties of the cartilage matrix, their function is disrupted. In early-stage, chondrocytes are trying to restore lost aggrecans by increasing their synthetic activity. Aggrecan degradation is first seen in the superficial layer, presenting as superficial cartilage fibrillation [50]. Another family of matrix-degrading enzymes, matrix metalloproteinases (MMP), participate in matrix degradation and OA pathogenesis [51]. MMP-13 is the most expressed proteinase in OA [52]. Its function, but also of other MMPs such as MMP-1 and MMP-3, is a disruption of the collagen network. Although MMP-13 can degrade a number of collagens and even aggrecan and other proteins, the primary target of MMP-13 is type II collagen, which is the most common type of collagen in the cartilage structure [52]. Perturbed equilibrium between anabolic and catabolic processes leads to the progression of OA and further structural changes [4]. The main mediators of cartilage metabolism are cytokines, discussed below.

Following the changes in the ECM, the pericellular matrix also undergoes degradation by increased proteinase activation. During the repair of cartilage matrix damage induced by excessive mechanical loading, chondrocytes gather themselves in clusters to further increase their synthetic activity [31,53]. To maintain high metabolic activity, chondrocytes experience a proliferative response, but they also adjust by undergoing hypertrophic differentiation [30]. The generation of cartilage degradation products, together with the secretion of damage-associated molecular patterns (DAMPs), further increases the release of proinflammatory mediators. They also influence the adjacent synovium to proliferate and induce inflammatory responses, ultimately leading to larger decomposition of the cartilage [8].

By initiating inflammatory processes and inducing a catabolic state of the cartilage, early surface changes seen as fibrillations extend distally, forming deep fissures, leading to cartilage delamination uncovering the calcified cartilage and the subchondral bone [5,31,48]. The thinning of hyaline articular cartilage is accompanied by the expansion of underlying calcified cartilage, which additionally contributes to increased mechanical stress and the further production of catabolic factors [8]. Furthermore, an enlarged layer of calcified cartilage advances into the overlying AC together with a duplication of the tidemark as another finding in osteoarthritic knee joints. These changes are caused by the penetration of vascular channels from the bone marrow, through the subchondral bone, calcified cartilage and tidemark to the articular cartilage, accompanied by sympathetic and sensory nerves [54].

### 2.2. Subchondral Bone

Even though progressive cartilage damage and its eventual loss were the most mentioned features of the OA in the past, it is now well accepted that subchondral bone alterations and synovial inflammation also influence all other structures in the joint. Their structural changes are important disease characteristics, confirming that OA is a whole joint disease. Moreover, numerous studies suggest that interactions between cartilage and subchondral bone are fundamental for joint homeostasis, but also disease progression [30,31,48,55,56,57]. Relative to the slower turnover rate of the AC, subchondral bone undergoes more rapid modeling and remodeling in response to changes in the mechanical environment [55]. Subchondral bone consists of two layers: the plate-like layer of cortical bone beneath the calcified cartilage, also known as a subchondral bone plate, and the deeper layer of subchondral trabecular or cancellous bone [56,57]. The structure of subchondral bone is mostly dependent on two types of cells that synthesize new bone and resorb the old one in response to the local environment—osteoblasts and osteoclasts [57]. A study by Sanchez et al. reported that osteoblasts reply to mechanical stimulation, just like chondrocytes, by adapting their metabolic activity and secret pro-inflammatory cytokines and degradative enzymes [58].

In established OA, the subchondral bone plate increases in volume and thickness. These changes are accompanied by alterations in subchondral trabecular bone in terms of its deterioration in the early stage and sclerosis in the late stage of OA [59]. Furthermore, modifications of osteoblastic and osteoclastic activity result in bone turnover, causing subchondral bone lesions, cysts and osteophyte formation (Figure 2) [60]. 

Bone marrow lesions (BML) represent micro-damage to the bone and are characterized by localized fibrosis, fat necrosis and a local increase in bone remodeling that results in microfractures of the trabecular bone [61]. These features are commonly associated with overlying cartilage erosion and fissures, particularly with exposed subchondral bone [62]. It has also been found that the appearance of BMLs and denuded areas of subchondral bone are related to clinical symptoms, particularly pain [57,63]. With the initiated increased bone turnover and neovascularization of subchondral bone, newly formed vessels together with nerves infiltrate the bone, invade the overlying cartilage tissue, making a communication channel for the exchange of biologic factors [57]. This process is another mechanism that causes pain in OA. Subchondral bone cysts development, a hallmark of the advanced OA, is dependent on osteoclast-mediated bone resorption, a process initiated by bone damage and necrosis at sites of former BMLs [64]. As an additional adaptive mechanism, osteophytes form at the joint margins via endochondral ossification [4]. A recent experimental rat model found that subchondral bone changes occurred before cartilage degeneration in ACL transection, while cartilage changes occurred before the subchondral bone changes when collagenases were injected into the joint, suggesting a different pathophysiological response in primary and secondary OA models [65]. Although the aforementioned subchondral bone structural changes are associated with subsequent cartilage loss, there is no correlation with subchondral sclerosis detected by MRI, signifying sclerosis is a late-stage characteristic of OA [66].

### 2.3. Synovium

The synovial membrane, together with synovial fluid, makes the synovium [67]. Synovial fluid plays a crucial role in cartilage nutrition. The avascular cartilage uses the synovial fluid as a source of nutrients, but also as a reservoir for its degrading products [68]. The synovial membrane consists of intima (a lining layer) and subintima (a sublining layer) thick up to 5 mm in healthy individuals [69,70]. Two to three layers of metabolically highly active cells (synoviocytes) lie over vascularized loose connective tissue to form the subintima, with plenty of collagen secreting fibroblasts [69,70,71]. Two types of macrophages have been found in synovia: classic and inflammatory-like macrophages [72]. In knee OA, inflammatory macrophages are important mediators as they produce VEGF, which may be a possible mechanism causing synovitis and inflammation [69,73]. Macrophage-like synoviocytes are CD163- and CD68-positive, but also stain positively for nonspecific esterase, an enzyme histochemical stain that differentiates macrophages and monocytes from other cell types. They form an integral part of the intima, together with fibroblast-like synoviocytes, which are CD55-positive and express class II major histocompatibility molecules [69,70]. Macrophage-like synoviocytes proliferate during inflammation, whereas fibroblast-like synoviocytes mainly produce hyaluronic acid and are found further from the intima [70,71]. The synovium produces hyaluronan and a plasminogen activator, preserving synovial fluid volume during exercise. It also secretes lubricin and hyaluronic acid, important synovial fluid components [73]. 

Synovitis is recognized as an important feature in patients with OA and has been associated both with symptoms and with structural progression. The inflammation in OA causes synovia to proliferate and induces the infiltration of T and B lymphocytes as well as mast cells [74,75]. Synovial hypertrophy is defined as a synovial thickening of ≤4mm and effusion depth of fluid ≤4 mm or ≤4 mm in the suprapatellar recess [76]. Stimulated by mediators of inflammation, particularly Interleukin-1 (IL-1) and Tumor Necrosis Factor (TNF), MMPs and other proteinases induce chondrocytes, synovial cells and lymphocytes to produce IL-6, IL-8, IL-15, IL-17, leukemia inhibitory factor and prostaglandin E2 (PGE2) [70,77,78]. Studies demonstrated that MRI confirmed synovitis strongly correlates with pain severity expressed by the WOMAC pain score [79]. In addition, synovitis causes the extensive production of proteolytic enzymes, causing cartilage damage, while cartilage matrix catabolism produces molecules that propagate synovial inflammation [71].

Synovial thickening is associated with radiographic and clinical progression of knee OA, pain and dysfunction that predominantly occurs posterior to the ACL and in the suprapatellar region [80]. It is commonly identified during arthroscopy in approximately 50% of patients with OA [81]. Moreover, areas of inflamed synovium tend to relate to locations of cartilage degradation [76]. However, in advanced OA, synovitis is diffuse, especially after total knee arthroplasty [74]. Because of the increased peripheral nociceptive neuron responsiveness in synovitis, OA symptoms and pain sensitivity are progressive [82,83]. A large study by The European League Against Rheumatism (EULAR), which included 600 people with knee OA, demonstrated synovial hypertrophy or the effusion in 46% of patients [84]. A study that evaluated the frequency of synovitis in OA knees using non-contrast MRI to assess synovial thickening showed that synovitis (defined as synovial thickening) was observed in 73% of OA knees compared with 0% of the control group. Synovitis was noted to be more likely to be present with increasing Kellgren–Lawrence (K/L) grade [85]. The synovial thickening observed on MRI has been confirmed as histological synovitis, with the grade of thickening correlating with the degree of macroscopic synovitis seen at arthroscopy and microscopically. The authors also noted that the distribution of synovitis was diffuse, with no statistical difference seen between those patients with marked chondral changes and those with few chondral changes, suggesting that synovitis is present from the earliest stages of OA and is diffuse and not related only to areas of cartilage [85,86]. Contrast-enhanced MRI may better differentiate inflamed synovium from joint effusion, but it is still not known whether contrast-enhanced MRI assessment of synovitis can predict disease progression in OA [76].

### 2.4. Infrapatellar Fat Pad, Menisci, Periarticular Muscles, Ligaments and Tendons 

As the largest intraarticular adipose structure, Hoffa’s infrapatellar fat pad (IPFP) is located in the anterior knee compartment, between the synovium and the joint capsule [87,88]. As a shock absorber, Hoffa’s fat pad acts to reduce the impact of loading forces, protecting the knee from mechanical damage [88,89]. IPFP is a very sensitive tissue containing adipocytes, fibroblasts, leukocytes, macrophages and other immune cells [88]. It is innervated by C-fibers that release substance P. Increased concentration of substance P is present in patients suffering from OA. Vasodilatation and migration of immune cells result in swelling, local ischemia and partial necrosis due to an increased concentration of substance P, causing structural changes in IPFP [88]. Macrophages found in the IPFP of patients suffering from OA are increased in number and similar to macrophages of the synovium and subcutaneous fat tissue with CD11c+ and CD206+ markers [90]. Hoffa’s fat pad is a potent producer of adipokines and cytokines such as adiponectin, leptin, IL-6 and TNF, which makes IPFP an active endocrine organ [89,91]. In OA patients, it is reported that IPFP and synovial fluid contain significant amounts of FGF-2, VEGF, TNF α, and IL-6 [92]. Moreover, the severity of knee OA seems to be associated with the concentration of leptin in the synovial fluid [93]. Collectively, alterations to Hoffa’s fat pad may be a possible cause of chronic knee pain in OA [88]. 

One of the strongest risk factors for developing knee OA is meniscal lesions and injuries, due to decreases in resistance to mechanical forces, such as tension, compression and shear stress [94]. Loss of intact meniscus function leads to OA due to joint instability and abnormal mechanical loading [95,96]. Moreover, in patients with radiographically confirmed OA, meniscus damage is almost always present, causing loss of load-bearing, shock absorption, lubrication and instability, which can lead to static changes in the femorotibial joint, leading to damage in the AC, as well as in the subchondral bone, contributing to the progression of knee OA [94,95,97]. In healthy individuals, the collagen matrix in loaded meniscus resists biomechanical stress, allowing load transmission to other knee structures, making the knee stable during movements [98]. If the meniscus is injured or damaged, it often results in damage to other knee structures, such as cartilage loss, changes in subchondral bone, lesions of bone marrow and synovitis [99]. It has been demonstrated that meniscal subluxation and extrusion are an independent predictor of tibiofemoral cartilage loss and degenerative subchondral bone marrow lesions [100]. Patients with mild and moderate knee OA showed significantly increased meniscal tear and extrusion [101]. The history of partial or complete meniscectomy is also a risk factor associated with the incidence of OA of the knee [102]. In summary, loss of meniscal function leads to OA and vice versa [94].

One of the main functional limitations in knee OA is the dysfunction of the quadriceps muscle, hamstrings and hip muscles [103,104]. The weakness of periarticular muscles has a significant impact on knee biomechanics. However, it is not clear whether muscle weakness is associated with OA onset or OA progression [105,106]. Increased intramuscular fat content in the quadriceps was found to be a strong predictor of knee cartilage loss [107,108]. It was also noted that periarticular muscles in patients with OA are inflamed and are producing myokines [105,109]. Observing the molecular aspect, myokines interact with synovia, Hoffa’s fat pad, cartilage and bone, making skeletal muscles involved in OA pathogenesis [109]. Tendon instabilities and ligament injuries have also been recognized as predisposing factors of OA, with around 50% of patients developing OA after ligament injury in a period of 10-20 years [91]. Studies demonstrated that neuromuscular training can decrease the possibility of developing knee OA after previous knee injury [106]. In a 10-year follow-up period, anatomic ACL reconstruction was found to reduce the risk of post-traumatic OA development [110]. The importance of knee muscles and ligaments in everyday functional actions, such as standing up from a chair, going up and down the stairs and level surface walking is evident [105]. In patients with symptomatic knee OA, muscle size maintenance is associated with beneficial structural changes and a reduced risk of knee joint replacement [111].

### 2.5. The Role of Cytokines, Chemokines, Mirna, Gene Expression and Other Molecules

#### 2.5.1. Cytokines Involved in OA Signalization

Our understanding of OA pathogenesis is incomplete without cytokines. Today, we know that OA is not merely a condition that develops from inappropriate loading forces on weight-bearing joints and repetitive stress, but a disease in which an underlying immune response dictates the degradation of cartilage, bone remodeling and typical symptoms such as swelling, pain and stiffness [112,113,114]. How this inflammatory response is initiated is unclear, but it is known that joint tissue cells can produce and respond to inflammatory stimuli [114]. In animal models, as well as in studies observing human OA, an imbalance between inflammatory and anti-inflammatory processes has been observed, causing a perpetual underlying low inflammation state [115]. The proinflammatory state favors catabolism over anabolism in the joint, resulting in cartilage degeneration and destruction by impairing the balance that causes stable turnover of cartilage and the ECM [116]. The main signaling molecules of the immune response in OA are cytokines. Based on their effect on the metabolism in the surrounding tissue, they are commonly divided into two distinct subgroups: inflammatory and anti-inflammatory. The main inflammatory cytokines are: IL-1β, TNF-α, IL-6, IL-8, IL-17 and the main anti-inflammatory cytokines are: IL-1Ra, IL-4, IL-10 and IL-13 [2,77,117,118,119,120]. Il-1β plays an important role in knee cartilage. When stimulated with Il-1β, type IX collagen is torn on its chains. The cartilage releases COMP when stimulated with Il-1β, allowing the fragmentation of this molecule [121]. The primary effect of IL-1β and TNF-α in OA pathogenesis is the perpetuation of the inflammatory response by induction of the cascades regulating the expression of important pathway genes, such as those of the NF-kB, ERK, JNK and p38 kinase. They also increase the synthesis of prostaglandin E2 (PGE2) and induct synthesis of ADAMTS in chondrocytes [2,77,113,114,117,118,122]. The effect of NF-κB activation is considered the main catabolic regulatory genetic pathway in chondrocytes [113,114]. The activation of NF-κB promotes the production of hypoxia-inducible factor 2 α (HIF2α), nitric oxide synthase (NOS2), COX2 and IL-1, which promote the inflammatory process, induce MMPs and ADAMTS, and self-perpetuate this mechanism by the synthesis of IL-1 [4,114]. IL-6, IL-8, IL-17 and IL-18 synthesis is also induced by IL-1 [2,114,123]. Hypoxia-induced factor 2 (HIF2α) induces the expression of matrix-degrading enzymes as one of the catabolic factors involved in the pathogenesis of knee-OA-enhancing chondrocyte apoptosis [124]. The inflammatory effect of IL-1 is mitigated by IL-1Ra with its concentration being higher in OA patients when compared to concentrations in healthy controls [113]. Endogenous molecular mechanisms that down-regulate the effect of IL-1 and TNF-α are also activated in OA. Their effect has been shown to reduce the progression of cartilage loss in OA models [77]. Another distinct effect of IL-1β and TNF-α is the promotion of chemokine synthesis, namely IL-8, monocyte chemoattractant protein 1 (MCP-1) and chemokine ligand 5 (CCL5), which act as mediators between local tissue inflammation process and the effect of immune cells [125]. IL-8 acts as both a cytokine and chemokine. Its effect is similar to that of IL-1: the induction of NF-κB, suppression of chondrocyte proliferation and promotion of metalloproteinase synthesis. It serves as a chemokine by attracting and activating neutrophils. Measurements of IL-8 levels in the synovial fluid have shown that it correlates well with OA grade and MMP-13 activity, while a decreased concentration of IL-8 has been observed following anti-inflammatory therapy [117,120,122].

IL-6 is also observed to mediate the inflammatory process in OA. It promotes the inflammation and synthesis of MMPs by activation of JAK/STAT pathway when it binds to IL-6 receptors, found not only on the chondrocyte membrane but also in the synovial fluid as soluble receptors, whose shedding has been correlated to cleavage by ADAMTS [126]. In experimental models, IL-6 has been found to promote the production of MMP-3, MMP-13 and ADAMTS, whilst its inhibition has been observed to alleviate the progress of OA lesions [127]. Higher MMP-9 and IL-6 levels were demonstrated in the analysis of the chondral tissue of OA patients [128]. A positive feedback loop between IL-6 production and calcium phosphate crystals appears to promote OA progression, as found in in vitro studies [129]. Interestingly, the production of IL-6 by synovial fibroblasts has been observed to be greater in obese patients [130].

Together with cytokines, activated complement factors are important in the pathogenesis of knee osteoarthritis. There are assumptions that molecules such as fibromodulin and other small leucine-rich proteoglycans (SLRP) molecules drive the inflammation targeting the classic complement pathway in cartilage. Moreover, cartilage oligomeric matrix protein (COMP) and the C3b component of the complement, which activates a local immune response in the joint, are found in the synovial fluid [130]. 

Proteomic analysis of 92 serum markers in OA patients compared to healthy controls found that there are several cytokines in OA with low expression levels, such as Caspase-8 (CASP-8), Extracellular Receptors for Advanced Glycation End-products (EN-RAGE) and Delta and Notch-like epidermal growth factor-related receptor (DNER), which stimulate the immune response and inflammation. Deletion of Axis Inhibition Protein (AXIN1) leads to OA degeneration. Blocking the function of STAM-binding Protein (STAMBP) decreases the level of IL-1β, one of the most important inflammation cytokines. Furthermore, decreased levels of SIR2-like Protein 2 (SIRT-2) are reported to play a role in inflammation cascade and pain development. Stem Cell Factor (SCF) downregulation was observed in OA patients, indicating its potential involvement in pain regulation of OA. Decreased serum levels of Eukaryotic Translation Initiation Factor 4E-Binding Protein 1 (4E-BP1) correlate with pain intensity. Increased levels of IL-6, CSF-1, FGF-21, Monocyte Chemotactic Protein 3 (MCP-3), known as CCL7, and TGF-β1 have been found in the serum of patients with KOA [131].

Another important chemokine in OA pathology is VEGF. Produced by hypotrophic chondrocytes, macrophages and synovial fibroblasts, VEGF has been observed to inhibit the synthesis and expression of aggrecan and type II collagen in the ECM [132,133]. The inflammatory properties of VEGF reported in other tissues have not been reported in OA studies [113].

Transforming growth factor β (TGF-β) secreted in its inactive form into the ECM has an important role in cartilage and subchondral bone metabolism. In the case of tissue injury, it is activated and acts as a signaling molecule for reparatory stem cells. This process activates both in trauma and in normal bone remodeling when osteoclasts resorb the bone. Its effect is paramount to normal bone and cartilage turnover knowing that mutations causing the premature activation of TGF-β are the key pathogenic factor for developing certain skeletal deformities [55]. In healthy cartilage TGF-β has an anabolic effect and induces proliferation by the activation of ALK5 receptor, however, in OA it switches its receptor to ALK1, which in turn changes its function from anabolic to catabolic by a change in the signaling cascade [134]. TGF-β1 found in the synovial fluid of OA patients induced the expression of MMP-3, MMP-13 and IL-18 receptors in chondrocyte culture, whilst in a rat model, the formation of a chondrocyte aggregated structure was observed in addition to the aforementioned effect [135]. In vitro models have demonstrated that TGFB1 produced by MSCs has a pro-anabolic function on chondrocytes that inhibits cartilage and bone degradation, it also inhibits calcification in the soft tissues [136]. Elevated levels of TGF-β1 in the subchondral bone were observed to promote osteoclastic activity, increase angiogenesis and promote proteoglycan loss. These observed effects were attenuated in rodent models by the inhibition of TGF-β1 [137]. Chondroprotective effects were also observed in rats with OA treated with glucosamine perorally, presumably by decreasing the levels of TGF-β1 and increasing those of IL-10 [138].

Due to their role in the pathogenesis of OA cytokines became molecules of interest as predictive biomarkers of OA. Studies have shown that the level of some of the aforementioned cytokines changes with the OA grade, suggesting their potential in the clinical setting. Neural network analysis of cytokine profile in the patient’s serum showed promise in the differentiation of OA, rheumatoid arthritis and healthy controls, indicating its potential use as a biomarker in the future to detect early OA before the typical radiographic features become apparent [139]. TNF-α, IL-1Ra, IL-4 and IL-8 correlate with OA grade when compared to radiographs [117]. Another open topic is a sex-related expression of cytokines. Further studies are warranted, as they could provide an explanation for the greater prevalence of OA in female sex [140]. Apart from their role in the inflammatory process in the joint, a correlation of pain and the concentration of cytokines has been recognized, with IL-6 and IL-8 showing a correlation with pain on movement, and TNF-α correlating with both pain on movement and rest [141]. Moreover, IL-10, IL-12, IL-13, VEGF, SCGF-β, fibroblast growth factor-21 and Eukaryotic translation initiation factor 4E-binding protein 1 levels were correlated with knee pain and functional impairment in an analysis of knee OA patients [131,142]. Studies comparing patients with knee OA and hip OA have demonstrated that the profile of cytokines differs not only among patients but among joints, suggesting that knee and hip OA have different cytokine release profiles and that hip OA measured higher concentrations of inflammatory cytokines when compared to the knee [143,144]. These results were reinforced in a study comparing the profile of cytokines in knee OA patients which showed different levels of inflammatory markers in unicompartmental OA when compared to bicompartmental OA [142].

#### 2.5.2. Adipokines 

According to The World Health Organisation, obesity is defined as abnormal or excessive fat accumulation that presents a risk to health. A person with a BMI greater than 30 is considered obese [145]. The prevalence of obesity is constantly increasing, reaching pandemic levels [146]. Obesity is a well-known risk factor for the development of OA, with the incidence of knee OA in obese persons four times greater than that in the control group [147]. The exact mechanism of how obesity increases the risk of OA is not clear. Some authors suggest that an increased risk of OA is connected with increased joint loading [148]. On the other hand, some studies showed that a combination of biomechanical and metabolic factors is responsible for the connection between obesity and OA of the knee [149]. Sharma and co-authors showed that obesity is associated with OA at non-weight bearing joints as well [150]. This comes as proof that another factor, other than increased loading, is the cause of OA. Studies are suggesting that adipokines play an important, but not yet fully understood, role in OA pathogenesis. Adiponectin, resistin, leptin and visfatin are adipokines associated with OA. Our current understanding of adipose tissue and its role in inflammation and inflammation-mediated disease provides new insights into the model of OA development. Systemic adipose-derived inflammation in OA seems to vary in impact level on different joints, which suggests that the paracrine effect of fat tissue in the joint is more important than systemic metabolic impairment [151]. Fibroblast cultures treated with leptin have expressed higher levels of IL-6, one of the main promoters of inflammation in OA [129]. Considering localized adipose tissue mediated inflammation, IPFP, in particular, was an object of interest in conducted studies, due to its intra-articular position. Studies observing IPFP were inconclusive to its exact effect in OA, but elevated levels of adipokines were isolated from the synovial fluid compared to the serum of OA patients, suggesting they have a role that remains to be defined [151]. Animal models showed that obese models with impaired leptin pathways do not develop OA of the knee, suggesting that paracrine and endocrine function of adipose tissue affects disease progression and modification [152]. Conservative treatment of OA includes lifestyle changes that aim to reduce the patient’s weight to relieve the loading forces on the affected joint. Studies on adipokines and obesity have demonstrated that patients who lose weight either by diet or diet and exercise modifications exhibit a greater clinical benefit than exercise on its own [151]. However, the exact mechanism explaining the observed clinical effect is elusive. Collins and co-authors published a study whose main goal was to quantify biomechanical and biochemical changes in tibiofemoral articular cartilage in patients with high BMI and increased body fat percentage. Using MRI examination, they concluded that obese, asymptomatic patients, without a history of knee injury or knee surgery, had a lower pre-exercise tibial cartilage thickness and higher tibiofemoral cartilage compressive strain following loading. They also found that patients with high BMI had increased tibiofemoral T1ρ relaxation times, which could be a sign of decreased proteoglycan content inside the cartilage [153]. In the most recent review paper, Boyce and co-authors concluded that the revision rate of total knee arthroplasty in morbidly obese patients (BMI higher than 40) is increased relative to non-obese (7% vs. 2%). The risk of perioperative complications (superficial wound infection and periprosthetic joint infection) was also higher in those patients [154].

#### 2.5.3. Genetics and Epigenetics of OA

A recent review points out that several genome-wide association studies have discovered 71 new genetic risk loci for OA development since the release of UK biobank genotyping data in 2017, increasing their number to a total of 90, confirming its polygenic etiology. The majority of these loci have small effect sizes, ranging from an odds ratio of 1.03 to 1.25 [27]. Out of the 90 genetic loci detected in multiple GWAS studies, 16 genes have been recognized as significant in knee OA. These genes are: *GDF5*, *ZNF345*, *SOX9/ROCR*, *SMG6*, *NF1*, *NFAT/WWP2*, *USP8*, *ALDH1A2*, *SBNO1*, *COL27A1*, *COL6A4P1*, *DUS4L/COG5*, *BTNL2*, *AP3B1*, *SDPR* and *LTBP1*. The predictive value of OA genetic risk loci variants could be a potential prognostic tool to predict OA incidence and progression, but, in our opinion, only if combined with other predictive OA factors, including relevant clinical information. On the other hand, OA genetic risk loci variants could help us in understanding the underlining biological mechanisms responsible for OA development. Eventually, that can lead to a potential target for OA therapy.

Another important factor to consider when developing genetic predictors is the epigenetic regulating mechanism (DNA methylation, histone modifications and non-coding RNA), which can result in a phenotypic shift in the chondrocytes, deregulation of the equilibrium between anabolic and catabolic events, and modification in gene expression of certain transcription factors, cytokines (proinflammatory or anti-inflammatory) and cartilage matrix proteins, eventually leading to OA progression [155].

Although the emphasis has been put on DNA methylation and mRNA interference, namely by non-coding RNA (miRNA), a recent breakthrough has been made in the research of histone modification in chondrocytes identifying 12 chromatin states [27]. In vitro studies reported that histone deacetylases (HDAC) suppress transcription factors important for chondrocyte hypertrophy and MMP-13 synthesis, and animal models have shown that endochondral ossification was impaired when HDAC3, 4, 5 and 7 were knocked out [28].

Our knowledge of DNA methylation of genes involved in OA is expanding. A number of genes involved in OA progression were found to be impacted by DNA methylation. The impacted genes include *SOX9*, *COL2A1*, *ACAN*, *MMP-13*, *GDF5* and *BMP7* [28]. Downregulation of DNA methyltransferase DNMT3B has been identified in animal models of OA and humans alike, while it’s overexpression in the same models was observed to have a chondroprotective effect, potentially identifying a target for gene therapy in the future [28]. A study by Shen et al. observed that the targeted deletion of *Dnmt3b* in transgenic mice articular chondrocytes results in osteoarthritis development at an early age. Furthermore, it was indicated that inflammatory signals play a role in decreasing DNMT3B levels, ultimately leading to alterations in cartilage metabolic pathways [156]. The effect of sirtuins (SIRTs), which are histone deacetylases (HDACs), on MMP-1 and MMP-13 expression is in line with these findings, suggesting another epigenetic mechanism of OA pathogenesis [28]. HDACs can also affect certain pathways and transcription factors, such as runt-related transcription factor 2 (RUNX2), which mediates MMP-13 expression in osteoarthritis. Cao et al. reported that decreased HDAC4 was associated with increased *Runx2* [157]. Following that, it was shown that the expression of matrix-degrading enzymes (MMP-1, MMP-3, MMP-13, ADAMTS-4 and ADAMTS-5) was increased. In addition, the simulated increase in HDAC4 lowered the mRNA levels of *Runx2*, *MMP-1*, *MMP-3*, *MMP-13*, *ADAMTS-4* and *ADAMTS-5*, decreased *IL-1β* expression, but increased the mRNA of type II collagen and aggrecan expression [157].

Many OA risk loci modulating the expression of target genes are located in the non-coding regions of the human genome. Evolving genomic techniques such as Assay for Transposase-Accessible Chromatin using sequencing (ATAC-seq), genome editing, and single-cell analysis, significantly contribute to understanding epigenetic influence on OA development. Recent evolutionary analysis, studying risk variants at chondrocyte chromatin datasets located at non-coding sequence near chondrocyte genes, showed that those loci are becoming optimized for both human and mouse developmental samples during knee evolution [158]. By using the ATAC-seq, Richard and colleagues showed that accessibility differences in knee development and later in life are influenced by separate regulatory (causal) enhancer variant (rs6060369) located in a classic BMP locus involved in knee development, *Growth Differentiation Factor Five* (*GDF5-UQCC1*) that impacts mouse knee-shape and osteoarthritis [158]. Across Eurasia, the “T” allele frequency at rs6060369 is between 40–70% and confers a 1.3-1.8-fold increase risk for OA [159,160]. Likewise, by mapping the chromatin regulatory regions (promoters and enhancers) applying ATAC-seq on the knee joint cartilages from OA patients, Liu and colleagues investigated enhancers’ alterations associated with OA. During the study, the authors have verified 7 OA associated SNPs (rs10851630, rs10851631, rs10851632, rs12905608, rs12910752, rs4238326 and rs35246600) reside in three chondrocyte-accessible enhancers of the predicted target gene ALDH1A2 [161].

All of the studies mentioned above give significant value to our understanding of the complex OA pathogenesis. Nevertheless, limitations of these studies are related to them being done on isolated cell culture lines and knockout animal models that do not necessarily have to exhibit the same effect in the clinical setting. Translational studies are needed in the future to better understand their implications in OA patients.

#### 2.5.4. The Role of miRNAs in Epigenetic Regulation of OA 

One of the epigenetic mechanisms regulating gene expression is non-coding RNA (ncRNA) [27]. miRNAs are a family of non-coding RNAs that bind to mRNA and regulate gene expression by modifying protein synthesis. This effect is caused by repression in translation or by induction of mRNA degradation [162,163]. It has been reported that miRNAs modulate several signaling pathways commonly active in OA, such as NF-κB, Wnt/β-Catenin, SIRT1/p53 and SDF1/CXCR4 pathway [163]. In OA cartilage, histone deacetylase and NAD-dependent deacetylase sirtuin are regulated by miRNA, together with transcription factor SOX9, important in the development of OA [163].

A large number of miRNAs have recently been identified in OA-affected tissues. Reports indicate that one gene may be regulated by several miRNAs, making miRNAs a hot research topic for the pathogenesis of OA [163,164]. A recent study compared the expression of miRNA and mRNA in healthy and OA cartilage in the same patient, identifying the miRNA interactome with 142 miRNAs and 2387 mRNAs, being differentially expressed [165]. In OA chondrocytes stimulated by IL-1β, two miRNA (miR-491 and miR-146a) were found as upregulating, whereas 42 miRNA were downregulating [164]. One miRNA has been reported to be significantly decreased in OA cartilage: miR-140 [164]. Among all the miRNAs involved in the epigenetics of OA, miR-140 exhibits the highest expression, regulating the homeostasis of cartilage, inhibiting and modulating inflammatory pathways [27]. Inhibiting ADAMTS-5 and MMP-13 indirectly by inhibiting IL-1, miR-140 mediates the degeneration of articular cartilage, and its mutation causes an autosomal skeletal dysplasia [28,164,166,167]. A similar effect was seen with miR-27a and miRNA-27b [113]. MiR-491, which is upregulated in primary chondrocytes, is noticeably changed as well [166]. MiR-455-3p regulates PAK2 (P21-activated kinase), whose levels are increased in OA cartilage, whereas miR-455-3p levels are decreased [168]. MiR-145 targets directly mitogen-activated protein kinase 4 (MKK4) and decreases the production of matrix-degrading enzymes triggered by TNF-α such as MMP-3, MMP-13, and ADAMTS-5 [169]. The overexpression of MKK4 induces TNF-α-mediated signaling pathway of activation and transcription of catabolic genes, which contributes to cartilage degeneration in OA [169]. miRNAs have been observed to regulate the inflammatory response in OA and modify cartilage metabolism. MiR-127-3p and miR-155-5p control the differentiation towards M1 macrophage phenotype, miR-241-5p, miR124-3p, miR-135-5p and miR146a-5p-5p towards M2 macrophage phenotype, and miR-21-5p both M1 and M2 macrophage polarization [170]. M1 and M2 balance is reported to play an important role in the pathogenesis of OA. Moreover, the ratio of M1 to M2 seems to be remarkably higher in knee OA [170]. The overexpression of miR-9, miR-98 and miR-146 reduced IL-1b induced TNF production in OA chondrocytes [171]. Furthermore, miR-99a-3p was reported to target 36 genes and be downregulated in patients with lesioned OA cartilage [27]. 

Loss of miR-204 and miR-211 function has been found to play an important role in whole-joint degeneration, stimulating articular cartilage destruction by inducing matrix-degrading proteases in articular chondrocytes and synoviocytes [172]. Contrary findings about miR-204 were published by Kang et al. who indicated that miR-204 is up-regulated in OA cartilage, where it interrupts proteoglycan synthesis, leading to matrix degradation. They found that antagonizing miR-204 regulates the disproportion of anabolic and catabolic events in the cartilage matrix, therefore, decelerating OA progression [173]. Interestingly miR-204 repressed GAG production by directly inhibiting mRNAs involved in cartilage proteoglycan biosynthesis, including genes crucial for chondroitin sulfate and hyaluronic acid formation. In vivo, intra-articular injections of a miR-204 analog exacerbated cartilage damage in the mouse model, while miR-204 inhibitor injection caused a reciprocal effect. Another finding of this research was that the injection of antisense oligonucleotides, which attenuate miR-181a-5p activity (which is increased in degenerated human facet joints and OA knee cartilage) reduced cartilage damage in knee joints while reducing catabolic gene expression and markers of cartilage damage. This finding was confirmed in human cartilage explants [27]. The observed effects of miR-204, which are seemingly opposite, can be interpreted by a different degree of potency of direct and the indirect effects of miR-204 on ECM components’ turnover. The indirect effect, mediated by matrix-degrading proteases in articular chondrocytes and synoviocytes, is obviously affected by other factors as well, thus having less dependence on miR-204. Conversely, the direct effect of miR-204 on proteoglycan biosynthesis could potentially be one of the crucial regulating factors in cartilage degeneration. However, further research regarding the role of miRNA in OA is needed to be confident about the function of each individual miRNA in OA pathogenesis and their combined effect on OA progression.

miRNAs are also subjected to regulation by common epigenetic alterations (DNA methylation, histone modification, and RNA modification), creating a complex feedback group with a major influence on final genetic expression. Studies are indicating that dysregulation of this looping effect may play an important role in the pathogenesis of various diseases, with OA being of no exception [174]. This complex microcosmos of epigenetic regulation remains to be investigated in the future before definitive conclusions can be drawn, however, the prospect of miRNA becoming a biologic marker for OA detection/progression or even therapy is exciting.

#### 2.5.5. The Mitochondrial Genetics and Epigenetics in Osteoarthritis

Dysfunction of the mitochondria has been reported in human chondrocytes in OA, due to decreased activity of the mitochondrial electron transport chain (MRC) and altered ATP synthesis. It is believed that there are two possible ways of disrupting mitochondrial function: somatic mutations in the mtDNA and a direct effect of cytokines, prostaglandins, reactive oxygen species and nitric oxide. Despite the fact that reactive oxygen species (ROS) are present physiologically in the body at nanomolar to micromolar concentration, their excess may cause damage to chondrocytes, bone and the surrounding tissue by oxidizing lipids and by altering DNA and protein structure [175].

Oxidative stress, the apoptosis of chondrocytes, cytokine-induced chondrocyte inflammation, calcification of the cartilage matrix may be influenced by mitochondrial dysfunction, leading to possible biomarkers for detecting early OA because of the polymorphisms in the mtDNA. The increase of chondrocyte mitochondrial mass has also been reported in OA and could be a compensatory mechanism because of the deficiency in the transfer of electrons and low ATP production [176]. Recent data are not only suggesting the profound influence of the mtDNA in the pathogenesis of OA but also on the nuclear DNA methylome of the chondrocytes [177]. Besides, authors indicate that mtDNA variants within mitochondrial cluster JT (human mtDNA haplogroup) act as a protective factor against the progression of OA in world populations. Modification of mitochondrial activity leaves an open space for future treatment options and a possible understanding of OA, which remains unclear.

## 3. State-Of-The-Art Non-Operative Therapeutic Consideration

Although knee OA is the most common progressive musculoskeletal disease affecting over 250 million patients worldwide, the main treatment has not changed much since 1968, when the first knee total knee arthroplasty was performed. Since then, joint replacement surgery has constantly been progressing. A good example of this process is the partial knee prostheses which can replace only the most damaged part of the knee, thus avoiding potential procedural complications, among other issues. Joint replacement surgery is still the gold standard in the surgical treatment of knee OA. However, it is believed that knee arthroplasty is the defeat not only of orthopedics, but of medicine in general, due to the fact that modern medicine still cannot find the answer that would stop the progression of OA in its early stages in order to avoid surgery altogether. Therefore, a lot of research effort has been put into discovering and a better understanding of its pathogenesis, genetics and biomarkers. These new findings have provided the clinicians with new biological treatments for OA that have the potential to slow down the progression and change the natural course of the disease. Although these therapeutic options found their place in clinical practice, the rising number of replacement surgeries performed still demands the development of new therapies, or optimizations of the options we currently possess which are mentioned in the next chapter. Our understanding of OA as a multifactorial disease is going to be of the utmost importance when going forward in using non-surgical treatment options.

### 3.1. Platelet-Rich Plasma

Besides their role in coagulation and hemostasis, many platelet or thrombocyte functions are related to tissue injury. Platelet activation results in the release of over 800 proteins and molecules contained in their cytoplasmic granules whose function is related to vasoconstriction, inflammation, immune reaction, angiogenesis, and tissue regeneration. Platelet-rich plasma (PRP) is defined as a volume of plasma with a platelet concentration several times higher than in peripheral blood. PRP is obtained by spinning a sample of the patient’s blood at high speed to separate red blood cells from the plasma. PRP affects synovial cells, such as synoviocytes and macrophages, endothelial cells, cells of innate immunity and components of metabolism in cartilage. The beneficial effect of PRP is mediated by chemokines, cytokines, growth factors, adhesive proteins, proteases and other small molecules, such as ADP, Serotonin, Calcium, Histamine and Epinephrine. In addition, PRP is safe, with no reported infections, worsened clinical features or serious complications. However, there are some adverse events reported due to repeated intraarticular injections of PRP, such as moderate pain, swelling and effusion for a couple of days [178]. There are four general categories of PRP based on leukocyte and fibrin content: leukocyte-rich PRP (L-PRP), leukocyte reduced PRP (P-PRP); leukocyte reduced or pure PRP, leukocyte platelet-rich fibrin and pure platelet-rich fibrin [179]. Although numerous studies confirm the impact of PRP on pain reduction in knee OA in the short- and medium-term (6–12 months), different modes of product preparation and application make it difficult to obtain conclusions regarding clinical results from such therapy [180,181]. Product characterization and dosage, as well as proper timing, treatment repetition period, location and application technique, need to be standardized.

New trends are emerging daily, which is best portrayed by the application of PRP intraosseously into the subchondral bone, one of the key pathophysiological components of OA pathogenesis, which was ignored in previous development of non-surgical treatment options. A most recent systematic review indicated the great potential of this approach, and a recent observational study demonstrated better outcomes at 6 and 12 months when PRP is applied both intraosseously and intraarticularly compared to the intraarticular application only [182,183]. The greatest limitation of these studies is the lack of consistent measurements of treatment outcomes that would enable more precise cross-referencing of study results [182]. 

Studies to date have focused on comparing the effects of PRP with various other preparations or procedures. It remains an interesting area of research for the application of PRP products combined with other procedures such as stem cell or hyaluronic acid application. The possible therapeutic potential of PRP products in OA is not fully investigated and utilized; further studies need to focus on these combined approaches.

### 3.2. Bone Marrow Mesenchymal Stem Cells

As discussed throughout the review, OA affects the entire joint. Inflammation-mediated factors produced within different joint structures and alterations in the function of immune cells and mesenchymal stem cells within the joint environment are important contributors to osteoarthritis [15,184,185].

Mesenchymal stem cells (MSCs) are specialized precursor cells, found in various tissues that retain the ability to self-renew and can differentiate with appropriate biological signals into different tissue-specific adult cells. In that way, MSC can replace aged or damaged cells, and thereby potentially maintain the function of the organ or tissue in the adult [186]. As chondrocyte are the only cells present in the articular cartilage, the potential of MSCs to differentiate into adult cells makes them a potential therapeutic modality for OA treatment. To date, this effect has been observed solely in in vitro, however, their alternative effect observed in injured tissue is to secrete immunomodulatory and trophic signaling molecules that prevent an over-aggressive immune response and promote local regeneration by secretion of anti-apoptotic, anti-scarring, angiogenic and mitotic signaling molecules. Additionally, they inhibit bacterial growth by secreting LL-37 [187]. It is because of these paracrine effects that they express, and the lack of evidence for in vivo differentiation of MSCs into chondrocytes, that a new term was coined for reference to these cells—medicinal signaling cells—keeping their original acronym, but indicating a new understanding of MSC function in the treatment of OA patients [186,188].

Native articular cartilage has a limited capacity for self-renewal. Because of their regenerative and immunoregulatory properties, MSCs have attracted great interest in OA treatment. MSCs’ in vitro ability to differentiate into a variety of cell types, including chondrocytes, was considered crucial for their application. In the course of OA, MSCs accumulate in joints and adjacent bone marrow lesions. This suggests that they may play a natural role in response to joint injury or pathology, but recent evidence suggests that the mechanism by which stem cell therapy may be effective in osteoarthritis remains unclear [7,189]. The recognized mechanisms by which MSCs possibly influence the restoration of lost cartilage volume [190] include:

1. Enhancement of natural repair mechanisms through the secretion of anti-scarring (KGF, SDF1, MIP1a, MIP1b), anti-apoptotic (STC-1, SFRP2, TGFB1, HGF), angiogenic (VEGF, TGFB1) and mitogenic (TGF-a, TGF-B, HGF, IGF-1, FGF-2, EGF) factors supporting other repair cells [191,192];
2. Interference with the underlying pathological or inflammatory process via immune modulation [193]. This effect is expressed through the suppression of Th1 and up-regulation of Th2 cells, inhibiting IFN-y production by interaction with NK cells, switching the phenotype macrophages from proinflammatory M1 to M2, and reducing the antibody production of B-lymphocytes [192];
3. MiRNA secretion, regulating the gene expression of the surrounding cells [192].

However, it is not known whether the injected cells function in vivo as precursors for articular chondrocytes or whether they express therapeutic benefits via mechanisms unrelated to direct production of cartilage. Furthermore, there could be more mechanisms of MSCs involved in cartilage regeneration that are not yet identified, so further studies are essential for their recognition as a viable OA treatment option. It seems that MSCs promote cartilage repair via paracrine signaling mechanisms and the secretion of soluble trophic factors. This was observed in a study by Wang et al., in which MSC-derived exosomes induced chondrocytes to synthesize type II collagen and decrease production and expression of ADAMTS-5, providing a stable ECM and reducing the turnover rate of surgically induced OA in mice [194]. These factors enhance cellular regeneration and induce bone formation by stimulating the proliferation and differentiation of endogenous semi-like progenitors found in most tissues and by decreasing OA inflammatory and immune reactions [191]. On the other hand, there are some studies showing that intra-articular use of MSCs for the repair of the cartilage is questionable, due to insignificant pain relief and functional improvement in patients with knee OA [195]. MSC can be found in nearly all tissues, and are mostly located in perivascular niches when pericytes are actively transformed into MSCs [196,197]. The bone marrow is the first investigated and an excellent source of stem cells (BM-MSC) [198]. Autologous BM-MSCs are safe at all tested doses; clinical trials of BM-MSCs have reported efficacy at higher doses ranging from 25 × 10^6^ cells and 40 × 10^6^ cells [199,200,201]. BM-MSC treatment in patients with OA results in an overall improvement in pain and symptoms and reduces synovial inflammation, but there is still an inconsistency in the literature related to the cartilage-regenerative ability of BM-MSCs [199]. These results were reinforced by a recent meta-analysis including 724 patients, which indicated improvement in pain level measured by Visual Analogue Scale (VAS), International Knee Documentation Committee (IKDC) function score, Tegner Activity Scale and Lysholm Knee score when compared to respective results before treatment with BM-MSCs [202]. There are also some therapeutic possibilities combining MSCs with biodegradable materials that seem promising but need to be investigated [203]. It is possible that such regenerative effects are more likely to be observed in the earlier stage and that BM-MSCs have dominantly chondroprotective influence in OA [199].

### 3.3. Intra-articular Application of Autologous Microfragmented Adipose Tissue with Stromal Vascular Fraction

Researchers from St. Catherine Specialty Hospital, together with partner institutions, conducted four clinical studies over the last few years, investigating the use of autologous micro-fragmented adipose tissue (AMFAT) in treatment of knee osteoarthritis [204].

The purpose of the first clinical trial was to evaluate the effect of intraarticular injection of AMFAT on a series of 17 patients with late-stage knee OA (Kellgren–Lawrence grades III and IV) 12 months after application in two ways: indirectly using functional MRI assessment analyzing the glycosaminoglycans (GAG) content in cartilage by means of dGEMRIC, as well as the clinical outcome on the observed level of GAG using a standard orthopedic physical examination including VAS assessment [205]. In the trial, a standard lipo-aspiration technique was performed, and the harvested fat was introduced into Lipogems^®^ ortho kit (Lipogems International SpA, Milan, Italy) for the process of micro-fragmentation. The final product of microfragmented adipose tissue was applied intraarticularly into the patients’ affected knee joints. Twelve months after the treatment, but also on two time-points during the follow-up (3 and 6 months), pain estimates measured by VAS decreased significantly, both for resting and movement estimates. Furthermore, cartilage GAG content, measured by dGEMRIC index, significantly improved in 52.9% of measurements and deteriorated in only 11.2% of measurements, which would be a normal disease course for the late-stage OA. Concurrently, a second study was performed where functional scores were assessed at baseline and 12 months after the treatment [206]. In that study, seventeen patients (85%) showed a substantial pattern of Knee Injury and Osteoarthritis Outcome Score (KOOS) and Western Ontario and McMaster Universities Osteoarthritis Index (WOMAC) improvement, significant in all accounts. KOOS score improved from 46 to 176% when compared with baseline, WOMAC decreased from 40 to 45%, while VAS rating decreased from 54% to 82% (all *P* values were <0.001). Three patients (15%) received a total knee replacement and were not followed up.

Another study included classifying and describing cell types contributing to the effect of treatment [207]. A stromal vascular fraction from lipoaspirate (SVF-LA) and stromal vascular fraction from microfragmented lipoaspirate (SVF-MLA) samples were characterized, and the following population phenotypes were identified within the CD45− fraction: endothelial progenitor cells (EPC), endothelial mature cells, pericytes, transitional pericytes, and supra adventitial-adipose stromal cells (SA-ASC). The immunophenotyping profile of SVF-MLA was dominated by a reduction of leukocytes and SA-ASC, and an increase in EPC, evidencing a marked enrichment of the latter cell population in the course of adipose tissue micro-fragmentation. The fact that EPC outnumbered mesenchymal stromal/stem cells (MSCs) indicates their inevitable involvement in the observed effect of the SVF-mediated cartilage treatment.

The last published trial included ten patients (18 knees) suffering from knee OA grades III and IV. The effect of AMFAT intra-articular injection 24 months after the application was accessed by estimating pain level using VAS and analyzing GAG content in cartilage facets measured with dGEMRIC index [208]. Study results indicated that dGEMRIC index measurements decreased significantly 24 months after a single intra-articular injection of AMFAT in only 7 out of 76 joints facets, meaning that in the other 69 measurements, the expected GAG content reduction over the natural course of the disease was opposed. Together with these findings, estimated pain on VAS significantly decreased over the 24-month period, both in resting and movement. Taken together, these studies suggest that the application of autologous micro-fragmented adipose tissue with SVF in patients with knee OA increases GAG levels in hyaline cartilage, consequently reducing pain and improving movement abilities.

These results were partially corroborated by similar studies that reported very positive and promising results, including the lack of adverse events or worsening of the clinical symptoms, encouraging future studies. The procedure is safe for patients, minimally invasive, quick, one-step and economic, providing a low percentage of complications and great compliance [209,210,211,212,213].

Studies in animals suggest that the use of adipose-derived MSCs in combination with PRP shows superior results of ECM matrix composition in regard to GAG and collagen composition, compression strength and when compared to stand-alone treatment, indicating a benefit for patients when treated with this combination. In this model, inflammation was ameliorated by the combination, as well as with standalone treatment [214]. The safety of this combined approach was confirmed in a study of 91 patients with 100 percutaneous joint injections performed [215].

MSCs derived from bone marrow do not differ from those derived from adipose tissue as they share their pericyte origin, surface markers, gene expression and potential for differentiation. Another important consideration is the amount of MSCs that can be derived from different harvesting sites: a gram of adipose tissue provides about 300 times more MSCs than a mL of bone marrow aspirate [216]. Considering the difference in harvested volume, AMFAT appears to be a more favorable method [217]. When it comes to chondrogenic differentiation potential, BM-MSCs provide better results than AMFAT MSCs in vitro, however, the addition of growth factors, such as FGF and TGF, dismisses the observed difference [217]. To date, there are no trials comparing the results of BM-MSC and AMFAT directly, but a comparative review of the literature found that both provide an excellent safety profile and favorable patient outcomes based on perceived pain, joint function and OA progression [216]. The key considerations that can be drawn from the reviewed literature are that there is a need for a more structured experimental method regarding the application of MSCs, as reported by Awad et al. [202]. Another key point going forward is the standardization of applied doses and the determination of optimal MSC dose for various patient groups, possibly in regard to the phenotype of OA.

### 3.4. Extracellular Vesicles

Earlier we discussed the efficiency of mesenchymal stromal/stem cells (MSC) in cartilage regeneration. However, most of MSCs paracrine effects that play critical roles in tissue regeneration are related to the release of extracellular vesicles (EVs). The commonly used term exosomes represents the membrane-bound extracellular vesicles surrounded by a phospholipid bilayer that contain different cell-specific receptors, integrins, etc., which are important in cell to cell communication [218]. As the main component of MSC secretome, extracellular vesicles after entering the cells regulate gene transcription and the function of recipient cells [218,219,220]. A recent study analyzed 13 preclinical studies that used MSC exosomes for cartilage repair and showed that in most studies, exosome treated animals shown increased cellular proliferation, augmented matrix deposition, and better histological scores [221]. Some authors argue that, in comparison with MSCs treatment, exosome-based therapy is more sustainable, reproducible and safe, primarily because of reduced toxicity and immunogenicity issues [222,223]. Moreover, it has been reported that, together with different biomolecules, exosomes containing RNAs, DNAs, mRNAs, and a higher proportion of miRNAs, when compared to the level of miRNA in its respective parent cells [224]. However, the studies related to exosomes from the synovial fluid and chondrocytes are still limited, and we have yet to understand their role in OA.

## 4. Future Strategies for OA Management

As stated throughout this review, OA is a complex pathological entity with many underlying pathophysiological mechanisms that can induce cartilage destruction and generate low state inflammation in the joints, ultimately causing pain, stiffness, swelling and loss of function. An ideal treatment for OA does not exist at this time and a great number of patients will ultimately end up requiring joint prosthesis to treat the advanced OA. Treatment options that are in use today show promise but, due to multiple etiology of OA, a one-treatment-fits-all approach could turn out to be wrong in the future. Clustering the patients in specific subgroups based on their clinical, biochemical, radiographic and molecular characteristics could, in the future, prove to be the right course of action, as the clinicians would have better and more specific treatment options that target specific mechanisms in OA pathogenesis.

### 4.1. Phenotyping OA

There is a growing interest in dividing OA patients into subgroups or phenotypes to better understand the individual patient’s need. In a review by Dell’ Isola et al., six distinct knee OA phenotypes were identified through a synthesis of 24 individual studies [225]. The authors propose chronic pain, inflammatory, metabolic syndrome, bone and cartilage metabolism, mechanical overload and minimal joint disease phenotypes. In a more recent study by the same group, 599 patients were classified into these six phenotypes, showing a correlation between phenotype groups and pain, as well as physical function [226]. Additionally, a seventh phenotype, named complex knee OA phenotype, was observed for patients that do not fit into any of the above-mentioned phenotypes because their characteristics overlapped between them. The research of OA phenotypes is only beginning, but further studies are expected with excitement as they could provide answers to the best individualized treatment options, provide the patients and clinicians with a tool that could be used to give a prognosis, facilitate the decision on conservative vs. surgical treatment, and eventually induce the development of treatment protocols. Phenotyping the patients could also prove to be a key stepping stone in better drug prescription and the development of drugs that could be used in the treatment of OA, as reviewed by Van Spil and colleagues [227].

### 4.2. Human-Recombinant Fibroblast Growth Factor 18 (Sprifermin)

As the function of fibroblast growth factor 18 (FGF 18) has recently been discovered, it has opened novel treatment possibilities for stimulating chondrocyte proliferation, inducing type II collagen expression and matrix production [228]. The intra-articular application of human-recombinant FGF 18 (sprifermin) showed a beneficial effect on cartilage, increasing the thickness of cartilage and reducing the cartilage loss in vitro, in vivo and several pre-clinical and clinical trials in humans. Sprifermin treatment stimulated chondrocyte proliferation and matrix synthesis while suppressing matrix metalloproteinase production [229,230,231]. Similar results were observed by Gigout, who has shown that FGF18 in 3D chondrocyte culture, increasing the number of matrix-producing chondrocytes, improving the type II:I collagen ratio and stimulating human OA chondrocyte to produce a hyaline extracellular matrix [232]. Despite promising results, the application of sprifermin should be considered with caution because it is a currently non-approved drug under development for OA treatment [233].

### 4.3. Bone Morphogenetic Protein 7 (BMP-7)

BMP-7 is a member of transforming growth factor-β (TGF-β) superfamily, observed to possess anabolic and chondroprotective effects in vitro, providing a base for its therapeutic effect in human OA [234]. In a rabbit model of osteoarthritis, Hayashi and colleagues have shown that the intraarticular injection of BMP7 inhibited the progression of OA in a rabbit ACL transaction model of knee OA [235,236]. Furthermore, a Phase 1 double-blind, randomized, multi-center, placebo-controlled, single-dose escalation trial demonstrated its safety with no dose-limiting toxicity [237]. The results of further phases are still expected to determine the efficacy of BMP-7 in OA treatment [238,239].

### 4.4. Monoclonal Antibodies

As a relatively novel therapeutic method, monoclonal antibodies (mAb) have been already approved and used for more than 30 targets and diseases, including autoimmune diseases, cancer, asthma, gout and hypercholesterolemia. Monoclonal antibodies have target selectivity and less toxicity when compared with small molecules [240]. In the knee OA, mAbs have shown promising results on mice models and in vitro studies. Targeting of the VEGF monoclonal antibody, including MF-1 (mAb to VEGFR1) and DC101 (mAb to VEGFR2), showed modulatory effects in OA knee pain in mice when applied intraarticularly [241]. A potential therapeutic option for treating chronic pain, in patients with symptomatic knee OA, could be Tanezumab, a mAb that blocks the Nerve Growth Factor (NGF) from activating Tropomyosin-receptor-kinase (Trk) receptors on nociceptive neurons [242]. Among patients with moderate or severe knee OA, response to tanezumab compared to placebo showed significant improvement in pain scores and physical function [243]. Moreover, an additional recombinant, fully human, anti-nerve-growth-factor antibody Fasinumab also showed improvement in walking knee pain and WOMAC scores [244]. Besides, the human anti-nerve growth factor monoclonal antibody appears to be well tolerated [245]. Human mAb ADAMTS-5 showed slowed cartilage degeneration and osteophyte growth but did not affect subchondral bone sclerosis in mice that underwent surgery [246].

Research of immune response in OA made the development of a therapeutic approach that would inhibit the effect of specific cytokines, potentially halting OA progression, a possibility. Clinical studies are scarce, and although they do show good tolerability with an already described side-effect profile of mAb therapy, a low level of effectiveness was observed. Drugs of interest include Adalimumab, Infliximab and Etanercept that inhibit TNF-α, and Anakinra that inhibits IL-1 and IL-1Ra genetic therapy, that is in the preclinical stage on the animal model [77,239]. Anakinra demonstrated encouraging results in animals but failed to demonstrate the same results in human clinical trials, whilst TNF inhibitors might prove their potential in the treatment of an inflammatory phenotype OA in the future [239]. The discouraging effect of immunomodulatory therapy is presumably a consequence of underlying mechanical damage provoking the immune response in OA, unlike in rheumatoid arthritis, where it is the key pathological mechanism. 

Taken together, present data suggest that monoclonal antibodies may exhibit a favorable risk–benefit ratio considering future targeted therapeutic methods for OA. However, this approach to OA treatment needs clear indications that would be specific for individual patients, as OA is a multifactorial disease with a complex pathogenesis involving multiple tissues. The clustering of patients into the previously mentioned phenotypes has the potential to establish a more individual approach, but further studies are needed to confirm this presumption.

### 4.5. Gene Therapy of OA

The shortcomings of systemic drugs, such as side-effects and impossibility of reaching sufficient concentrations in the joint, rapid clearance of intraarticularly applied therapeutic agents, but also the fact that OA is a heterogeneous disease, have raised the interest of applying gene therapy methods in OA treatment. Gene therapy offers an ideal combination of locally administered therapeutics and long-term effects, and therefore could be a future answer to the OA management obstacles. 

In a study by Nixon et al., the use of a helper-dependent adenovirus (HDAd)-mediated intraarticular gene therapy approach for long-term expression of interleukin-1 receptor antagonist (IL-1Ra) was investigated in small and large animal models [247]. Since IL-1 is an inflammatory mediator involved in a number of catabolic processes in OA pathophysiology, the continuous prevention of binding to its receptors could offer a sustained symptomatic and disease-modifying therapy for OA. The authors reported non-significant improvement in cartilage status (cartilage volume and bone surface covered by cartilage) and prevention of osteophyte formation in mice treated with HDAd-IL-1Ra, whereas a reduction in symptoms, decreased level of synovitis and improved cartilage status were observed in a horse OA model.

Newly developed gene-editing methods, such as clustered regularly interspaced short palindromic repeat (CRISPR), CRISPR-associated (Cas) endonuclease system, also served as a therapeutic potential for the disease. CRISPR system consists of a Cas9 nuclease and engineered single-guide RNA (sgRNA), which guides Cas9 to induce a double-strand break in a target site. Subsequently, DNA is repaired either with non-homologous end joining (NHEJ) or homology-directed repair (HDR). NHEJ leads to the accumulation of small insertions and deletions (indels), often causing functional gene knockouts [248].

Zhao et al. performed research in which they investigated the loss-of-function of the genes encoding NGF, matrix metalloproteinase 13 (MMP-13) and IL-1β in an induced OA mouse model, both separately and simultaneously [249]. This study has shown that targeted CRISPR-mediated ablation of NGF significantly decreased the level of pain, but also induced progressive cartilage disruption and osteophyte formation. In addition, NGF loss-of-function was associated with the upregulation of cartilage-degrading enzymes such as MMP-13 and ADAMTS-5, as well as aggrecan degradation products. In contrast, the targeted ablation of MMP-13 and IL-1β both lead to reduced cartilage degradation, lessened synovial hyperplasia, and decrease in osteophyte development, but also to downregulation of catabolic enzymes involved in cartilage matrix deterioration. Interesting findings were seen when all the genes were inhibited simultaneously. Multiplex gene editing of NGF, MMP-13 and IL-1β generated the alleviation of pain, an effect seen in NGF ablation, but also decelerated OA progression, ameliorating all the structures involved in pathogenesis, which was confirmed histologically and radiographically. Reported results showed the potential of CRISPR/Cas9-based gene editing for OA treatment in the near future. However, the therapeutical use of CRISPR/Cas9 system is reduced because of the potential off-target effects, the immunogenicity of sgRNA and Cas9 and insufficient delivery method. These limitations account for all CRISPR-mediated treatments or experiments, postponing the usage of this method in clinical practice before these drawbacks are overcome.

## 5. Conclusions

Although OA is the most common progressive musculoskeletal condition, its pathophysiology is not yet quite understood. Complex pathogenesis, with a large number of mechanisms leading to the same outcome, makes OA a field of interest for many researchers. Recent discoveries of cytokines involved in OA signalization and the role of miRNA in epigenetic regulation of OA suggest a potential for cytokines and miRNA becoming biologic markers for the early diagnosis of OA, which could be a key factor for successful and well-timed treatment. Current biological treatment of knee OA, including PRP and MSCs, offers significant results in terms of clinical outcome, seen as a reduction in knee pain, but also in the increase in GAG content in hyaline cartilage after the intra-articular application of autologous microfragmented adipose tissue with stromal vascular fraction, which was demonstrated in clinical studies conducted in our and other institutions. Therapeutic options such as sprifermin, BMP-7, monoclonal antibodies and gene therapy offer promising solutions, but more clinical studies are needed to confirm the safety and efficacy of these methods. In conclusion, a lot of studies, from a multidisciplinary perspective are required to better understand the pathogenesis of this highly prevalent disease. Furthermore, future studies that would address certain knee OA phenotypes, using novel but proven therapeutic considerations in OA treatment, such as MSCs, and objectification of their results using a wide range of biochemical markers, could improve our knowledge about OA pathophysiology and take a step towards more individualized, patient-specific treatment of knee OA.
